# Education and Economic Factors Shape Clusters of Biosecurity Beliefs and Practices: Insights from an Exploratory Survey of Midwest U.S. Swine Producers

**DOI:** 10.3390/pathogens14111080

**Published:** 2025-10-23

**Authors:** Benti D. Gelalcha, Maurine C. Chepkwony, Cesar A. Corzo, Colin Yoder, Andres Perez, Maria Sol Perez Aguirreburualde, Dennis N. Makau, Michael W. Mahero

**Affiliations:** 1 Department of Biomedical and Diagnostic Sciences, College of Veterinary Medicine, University of Tennessee, Knoxville, TN 37996, USA; bgelalch@utk.edu (B.D.G.); dmakau@utk.edu (D.N.M.); 2 Center for Animal Health and Food Safety, University of Minnesota, St. Paul, MN 55108, USA; cmcherotich@gmail.com (M.C.C.); yoder065@umn.edu (C.Y.); aperez@umn.edu (A.P.); mperezag@umn.edu (M.S.P.A.); 3 Department of Veterinary Population Medicine, College of Veterinary Medicine, University of Minnesota, St. Paul, MN 55108, USA; corzo@umn.edu

**Keywords:** swine farm, biosecurity, producers’ behavior, disease prevention, cluster analysis, machine learning

## Abstract

Despite existing biosecurity frameworks, there is limited empirical evidence on how US swine producers’ beliefs, behaviors, and risk perceptions influence enhanced biosecurity implementation. We conducted an online survey among US swine producers to understand their biosecurity beliefs, behaviors, and practices. We used descriptive, unsupervised machine learning, and Factor Analysis for Mixed Data (FAMD). Of fifty-four respondents, 48.1% reported implementing some biosecurity measures, and 72.2% valued having enhanced biosecurity protocols. Majority (53.7%) considered their veterinarian’s biosecurity opinion most important, and 37% were not concerned about African swine fever. Almost all (90.7%) felt confident they could contain an outbreak on their farms. However, none practiced enhanced biosecurity. The cluster analysis identified four distinct producer profiles (K = 4). Cluster A had young, inexperienced producers operating breeding facilities, with moderate biosecurity adoption. Cluster B included young, small-farm producers with variable biosecurity practices and low mortality rates. Cluster C comprised farms with moderate experience, higher mortality rates, and the lowest biosecurity adoption. Cluster D was composed of older, experienced, educated producers with the highest biosecurity standards and lowest mortality rates. FAMD revealed clustering along human capital and resource availability dimensions. Regular biosecurity assessments, tailored recommendations, and training would improve biosecurity in the swine industry.

## 1. Introduction

The United States (US) swine industry is one of the largest pork contributors to the global pork value chain, contributing approximately 12% of the world’s pork, estimated at more than 8 billion USD in export value [1,2]. As such, the swine industry is economically crucial in the US and globally. According to recent USDA census, there are about 75.1 million hogs and pigs on approximately 60,000 farms in the US. Majority (92.1%) of the 75.1 million hogs and pigs are market hogs, and only a fraction (8%) are kept for breeding. Iowa hog producers account for the largest inventory among the states, at about 33% of the total hog and pig inventory, while Minnesota has the second largest inventory at 12.3%. North Carolina is third with 10.3%. Most (75%) of these farms have more than 5000 head, although there are some (5%) smaller farms with less than 2000 head [3]. Due to its multi-site production [4,5] the industry is often challenged by various endemic viral and bacterial diseases, resulting in huge financial losses with untold negative social ramifications [6]. The reported economic losses from major swine diseases vary widely across countries and outbreak scenarios [7]. For instance, PRRSV (porcine reproductive and respiratory syndrome virus) costs the industry about 1.1 billion USD per year [8], a Foot and Mouth Disease Virus (FMDV) outbreak in the swine industry would result in losses between $57 billion and $200 billion, and an African Swine Fever Virus (ASFV) outbreak would cost about $50 billion [9]. Globally, the economic burden of these diseases is equally significant, with the estimated median cost of one or multiple co-occurring swine respiratory pathogen(s) ranging from $2.00 to $10.40 per nursery pig, $2.70 to $17.95 per fattening pig, and $117 to $378 per sow per year [10].

Progressively, the swine industry appears to have mastered how to mitigate the impacts of majority of these swine diseases through vaccination and the application of different biosecurity measures. However, the ever-present threat oof foreign animal diseases (FADs) such as ASF [11] necessitates regular evaluation and improvement of biosecurity measures.

Biosecurity, an essential component of successful intensive livestock production, such as in swine operations, involves a range of management practices designed to prevent the introduction and spread of infectious diseases [12,13,14]. These measures are essential for maintaining herd health, optimizing productivity, and ensuring a safe food supply [15]. In addition, investing in biosecurity provides significant economic advantages by reducing the risk of costly disease outbreaks, which can stem from mortality, morbidity, slower growth, and increased treatment expenses [16,17]. By improving and maintaining overall herd health, biosecurity measures also enhance production efficiency and help preserve the breeding stock’s value and genetic integrity [18,19]. In the US, enhanced biosecurity is a set of heightened disease mitigation measures designed to protect pork production from FADs and ensure continuity of business during an FAD incursion. It ensures that uninfected premises are kept safe in the event of an endemic or FAD outbreak [20]. These enhanced biosecurity measures consist of designating a line of separation, establishing controlled entry access points, Shower-in and shower-out, isolation facilities, age segregation, mortality management, air filtration, feed mitigants, transport biosecurity, and setting up cleaning and disinfection (C&D) stations to control access to the premises and isolate on-farm activity, in addition to having a well-thought-out biosecurity plan and a biosecurity manager to help lead its implementation [20,21,22,23].

Although the importance of biosecurity is widely recognized among all industry stakeholders, its implementation and compliance can be complex and challenging. Previous research in Europe revealed that implementation of enhanced biosecurity is affected by several factors such as perceived costs, evidence of efficacy and economic benefits, presence of economic incentives, producers, and farm workers levels of education and ongoing professional development, gender, farm size, production type, and the challenges of moral hazards and adverse selections often mediated by one’s risk perception [24,25,26,27,28,29]. Risk perception in agriculture refers to the subjective evaluation by producers /farmers of the probability of a known negative outcome and the uncertainty of the outcome [6]. However, there is a lack of empirical data on how these factors manifest among US swine producers, who operate under different structural, economic, and regulatory conditions.

Additionally, studies in the US have highlighted inconsistencies and complexities in adopting biosecurity recommendations, such as those outlined in the Secure Pork Supply (SPS) plan [15,20]. Understanding how producers perceive and implement biosecurity is key to developing feasible interventions and policies for real-world farm settings. This study aimed to characterize the beliefs, behaviors, and practices concerning the adoption and implementation of biosecurity measures by US swine producers on their farms. We hypothesized that the adoption of enhanced biosecurity practices is driven by modifiable attitudes and beliefs, with producers possessing varying capacities to implement such guidelines influenced by socio-economic and socio-cultural factors. Thus, we sought to assess producer attitudes and capacities regarding enhanced biosecurity implementation and identify critical areas for targeting improvement efforts. We employed a multifaceted approach: (1) generating new knowledge about the ability of swine producers to adopt enhanced biosecurity guidelines, while (2) disseminating information on practical ways to improve biosecurity.

## 2. Materials and Methods

We employed a mixed-methods approach to explore the beliefs, behaviors, and practices of farm biosecurity among swine producers in the US. The development of the survey and qualitative interview guide was informed by the theory of planned behavior [30,31] and the SPS framework (Supplementary Table S1). In this study, we adopted the social-cognitive theory, Theory of Planned Behavior (TPB), models that conceptualize beliefs about outcomes, social norms, and control as direct predictors of intention and behavior [30,31]. The Knowledge–Attitude–Practice (KAP) model posits a linear progression, it assumes that increasing knowledge leads to more positive attitudes, and subsequently to the adoption of desired practices, which is not always the case [32]. We therefore focus on producers’ beliefs and their actual behaviors/practices rather than on knowledge and attitudes. This approach is in line with established behavioral theories and indicates factors that are immediately actionable for devising interventions to improve biosecurity compliance.

This paper will focus on the quantitative results from the biosecurity survey disseminated between December 2021 and September 2022. We conducted the online survey using Qualtrics ^XM^ (Silver Lake Group LLC, Menlo Park, CA, USA) among 122 pig producers, primarily located in the Midwest U.S., with a smaller proportion from neighboring states. This region was emphasized because it represents the largest share of U.S. pork production, while participation from producers in other states offers additional perspectives. The sampling frame, therefore, captured a broad cross-section of swine operations, several of whom were part of large swine production companies that oversee between 50,000 and 250,000 sows per company, with concentration in the major pork-producing states. The questionnaire was developed based on established biosecurity recommendations outlined in the Secure Pork Supply (SPS) Plan [15,20] and informed by existing literature on swine biosecurity practices [33,34,35,36,37,38,39]. We distributed the survey through various online channels (to maximize reach and diversity of respondents), including the Center for Animal Health and Food Safety website, the Minnesota Pork Board e-newsletter, the University of Minnesota Swine Extension e-newsletter, conventional and non-conventional swine producer groups, and multiple extension social media platforms. We also distributed a postcard with a QR code to the survey directly to producers at the MN pork congress and the 2022 MN State Fair. While the dataset provides valuable insights into swine operations in this region, geographic concentration and the small size of the respondents may limit generalizability to other US regions with different production systems or regulatory environments. The anonymized self-administered online questionnaire, among other data, captured details on the experience of the respondents in swine production, perception of FAD risk/threat, and behavioral, normative, and control beliefs regarding biosecurity practices as prescribed in the SPS plan [15]. We conducted descriptive statistical analysis, including frequency summary statistics, to identify underlying trends among respondents using R statistical software (version 4.5.0; R Core Team, 2025).

We used cluster analysis to find naturally occurring farmer groups based on shared biosecurity attitudes, practices, behaviors, and influencing factors for precision intervention. Before running the cluster analysis, missing values in numeric variables were imputed with the median, and those in categorical variables with the mode. Some variables had missing values ranging from 9.1% to 32.7%, which were imputed using appropriate values. Furthermore, we conducted a multicollinearity test on the dataset before performing cluster analysis. We used pairwise correlation tests for continuous variables and Cramér’s V for categorical variables to identify predictors with strong correlations. The only significant correlation observed was between farmer age and farming experience, which is expected given the biological relationship between the two. Both variables were retained in the analysis given model performance and the fact that they offer distinct insights (age indicates demographic characteristics and generational influences such as technology adoption, and experience reflects professional background and the totality of their lived experience as swine producers). All categorical variables were converted to factors (i.e., category) data types, and numeric variables were standardized to ensure comparability across scales. Given the mixed nature of the data (continuous and categorical variables), we used the K-Prototypes technique to cluster the data [40]. The “kmodes” library in Python (v3.11) was used to implement the technique. For reproducibility, we set the random seed to 42 using the Cao initialization approach. Cluster (K) solutions were explored for K = 2 to K = 10 using internal validation metrics, the elbow method (to identify the reliability of clustering), and adjusted Rand index stability measures (ARI) (to identify the stability of clusters). A four-cluster (K = 4) option was determined to be the optimal partition based on these assessments. We characterized clusters based on the median values for the continuous variables and presented the distribution of the categorical variables as frequency (percentage) tables.

To further elucidate the potential underlying latent structure of the data, we performed Factor Analysis for Mixed Data (FAMD) using the Python package e “prince” (engine = ‘sklearn’). FAMD was selected based on the mixed nature of the data (continuous and categorical) and conducted on a subset of the dataset on five selected variables (farmers’ age, production experience, education, farm size, and operation type) guided by our knowledge and theoretical relevance (based on existing literature) of factors known to influence biosecurity adoption and compliance [20,41].

## 3. Results

### 3.1. Descriptive Analysis

The response rate to our survey was 44.3% based on the number of individuals who clicked on the survey link (*N* = 122). Therefore, for subsequent analysis, an *N* = 54 sample population (with complete data) was considered. The mean age of the respondents was 44.1 ± 12.9 years, with a median of 44 years and ranging from 15 to 64 years. These farmers had also been in swine production for 23.0 ± 14.8 years, with a median of 20 years, ranging from 2 to 58 years. More than half, 53.7% (29/54), of the respondents indicated that they had attained college-level education, and at least a quarter had a high school-level education. About two-thirds (68.5%, 37/54) of the respondents to the questionnaire were independent producers (of these, 13 had >500 swine), and about 22.2% (12/54) were strictly finish farms. Most 53.7% (29/54) of the responding operations had an inventory of 1–99 pigs, and 16.7% (9/54) had more than five thousand pigs.

**Figure 1:**
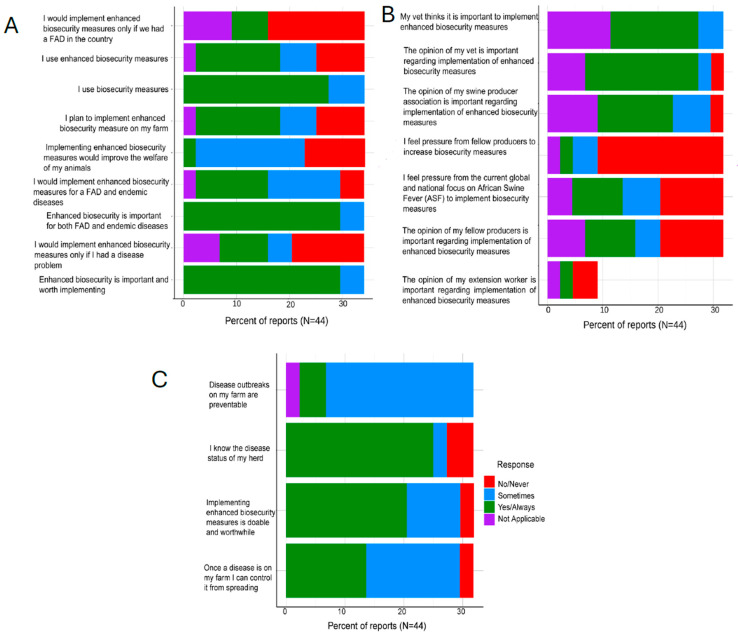
Swine Producer Biosecurity Perceptions and Practices. (**A**) shows perceptions toward implementing enhanced biosecurity measures on farms, including motivations, perceived benefits, and conditions under which they would adopt them; (**B**) shows the influence of veterinarians, producers, and external pressures on farmers’ decisions to implement enhanced biosecurity measures.; (**C**) shows farmers’ perceptions of disease prevention, knowledge of herd health status, and views on the feasibility of implementing biosecurity measures.

Overall, most responding operations reported that they always (48.1%, 26/54) or at least sometimes (33.3%, 18/54) implemented biosecurity measures such as LOS, having a perimeter buffer area, and ensuring their single feed trucks that serve multiple sites follow a biosecurity hierarchy. Similarly, all the responding operations believed that enhanced biosecurity was always (72.2%, 39/54) important and worth implementing or at least sometimes (11.1%, 6/54). Additionally, 50% (27/54) indicated they would use enhanced biosecurity if there was a FAD in the country (Figure 1A). About a third of the respondents indicated that their veterinarians (29.6%, 16/54) always thought it was important for them to implement enhanced biosecurity measures in their operations. The majority (53.7%, 29/54) consistently valued their veterinarians’ opinions regarding implementing enhanced biosecurity measures.

Most of the respondents (59.3%, 32/54) did not feel any pressure from fellow producers to increase biosecurity measures in their operations. Respondents had mixed feelings about how the global threat of African Swine Fever (ASF) influenced their operation’s biosecurity, with 20.4% (11/54) saying that they always felt pressure from the global and national focus on ASF to implement biosecurity measures, while almost twice as many (37%, 20/54) never felt the need to alter their operations (Figure 1B). Overall, most (68.5%, 37/54) respondents felt that they knew the health status of their herds well, all the time. Respondents felt that disease outbreaks were not always, but sometimes, preventable in their enterprises (59.3%, 32/54), similarly they believed that in the case of a disease outbreak, they were able, always (44.4%, 24/54) or sometimes (46.3%, 25/54), to control it from spreading (Figure 1C).

### 3.2. Cluster Analysis

The K = 4 clustering solution revealed distinct groupings among swine farmers, highlighting variations in terms of farmer demographics, farm size, disease risk, mortality, and biosecurity practices. The distribution of observations in the K = 4 cluster solution for swine farm biosecurity was as follows: Cluster A, *n* = 7 (smallest group); Cluster B, *n* = 17; Cluster C, *n* = 10; and Cluster D, *n* = 20 (largest group). 

Cluster A had younger (below average age), less experienced farmers (below average years in swine farming) with high disease risk perception for both endemic and foreign disease risk, but exceptionally low mortality. Farmers in cluster A can be labeled as ‘Young Risk-aware Breeders. Cluster A has a more balanced mix of contract growers and independent producers. This cluster is dominated by Sow/Breeding (71.4%) operations and has moderate adoption of biosecurity practices. Though lower in general adoption of biosecurity practices, farms in Cluster A responded Yes (71.4%) to “Daily” inspections of animals, which might help in early detection and intervention and a reduction in herd mortality. The farms in this cluster might be prone to high disease risk as they perceive, and target intervention measures needed to improve biosecurity practices and prevent disease introduction and spread in their farms.

Like Farmers in Cluster A, Cluster B farmers were younger and less experienced. But they had lower disease risk perception and reported a low mortality rate in their flock (Table 1). This cluster is dominated by small farms (82.4%) and independent producers (64.7%) with variable levels of biosecurity practices. Farmers in this cluster can be referred to as ‘Young Pragmatists.’

**Figure 2:**
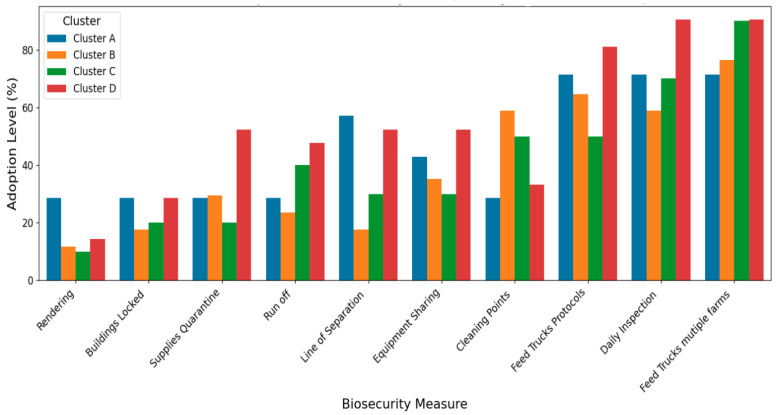
Biosecurity Measures Implementation Level by Cluster (ordered from Least adopted to Most). The Y-axis represents the percentage of respondents within each cluster reporting adoption of a given biosecurity measure (0–100%). The X-axis lists the specific biosecurity practices assessed. Cluster A shows moderate but consistent adoption across most measures, with strengths in line of separation and rendering. Cluster B exhibits the most variable adoption. Cluster C shows consistently low adoption across most measures. Cluster D demonstrates the highest overall adoption, particularly in resource-intensive activities such as “Feed Truck Protocols” and “Daily Inspection.”.

The low mortality in this cluster of smaller independent operations shows they managed disease risks adequately. Enhancing their biosecurity practices via continued support and training could help them prevent any local or foreign disease incursion and the spread on their farms. Cluster A producers had high disease risk perception and maintained moderate biosecurity implementation across most measures. Cluster A performs well in some operational practices, such as line of separation and rendering (Figure 2).

On the other hand, Cluster B producers, who were also young and inexperienced, demonstrated variable biosecurity adoption patterns, very low in some practices (e.g., locking of building and internal biosecurity, such as presence of a line of separation) and strong in others (e.g., presence of external biosecurity, such as cleaning points) (Figure 2). Farmers in this cluster have low disease risk perception and mortality. This group consisted of smaller operations that might have different considerations regarding biosecurity investments.

Farms in Cluster C, which were the ‘Complacent Independents,’ were characterized by moderate ages and production experience (Table 1). This cluster had 70% independent producers, 40% of whom had large farms, and 50% of them were college graduates (Table 2). It also had 20% contract growers and 10% extensive producers, along with a diverse operation size distribution (Table 3). This Cluster was characterized by consistently low adoption across most biosecurity measures, except for a few practices (Figure 2). The farmers in this group showed low disease risk perception, but the highest median mortality rate (0.95) among all other clusters (Table 1).

**Figure 3:**
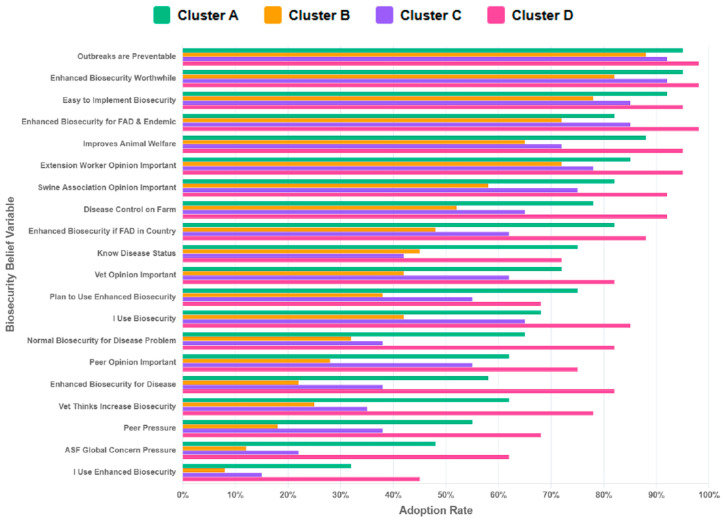
The distribution of beliefs about biosecurity measures across clusters (K = 4). The Y-axis represents the biosecurity-related belief questions (variables) posed to respondents within each cluster. The X-axis reports the proportion of farmers in each cluster adopting a given biosecurity belief (0–100%). Cluster A shows moderately positive beliefs across most measures. Cluster B shows variable belief patterns (some measures receiving firm support while others become weaker endorsement). Cluster C demonstrates weaker beliefs in most biosecurity measures. Cluster D demonstrates consistently strong beliefs across most biosecurity measures (mostly scoring above 60–80%).

Cluster D comprised older, experienced farmers, along with more educated individuals operating larger and mixed farm sizes (Table 1 and Table 2). They had a near-average perception of disease risk and experience below-average mortality rates (−0.51) (Table 1). Farmers in this cluster exhibited the highest adherence to biosecurity practices across all practices (Figure 2) and possessed strong positive beliefs about enhanced biosecurity (Figure 3). Farmers in Cluster D showed robust performance in the most critical and resource-intensive activities, such as Daily Inspection and Feed Truck Protocols. For example, when asked about their belief in enhanced biosecurity, over 95% of farmers in Cluster D responded “Yes/Always” compared to clusters with younger or less experienced farmers that reported lower levels of biosecurity measures. We referred to the farmers in this cluster as ‘Experienced Standard-Bearers.’

### 3.3. Factor Analysis for Mixed Data (FAMD)

**Figure 4:**
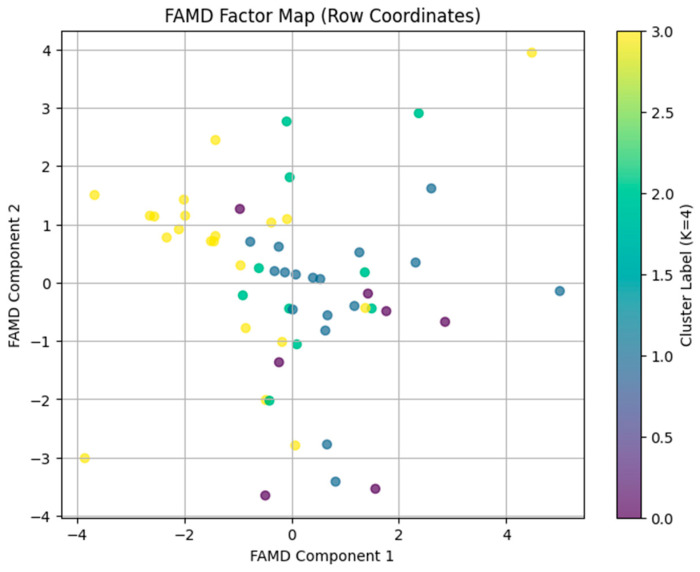
A scatterplot (the FAMD factor map) generated by plotting the factor scores for Component 1 (x-axis) versus Component 2 (y-axis). In this plot, each point on this plot represents a farm, and points are color-coded by their K = 4 cluster labels. Farms with similar profiles (in terms of age, production experience, education, farm size, and operation type) are located close together.

FAMD was conducted on selected continuous variables (farmers’ age and production experience) and categorical variables (e.g., education, farm size category, and operation type). The eigenvalues of the two FAMD components were 2.507 for Component 2 and 2.966 for Component 1 (Figure 4). These eigenvalues correspond to 45.80% (Component 2) and 54.20% (Component 1) explained inertia, respectively. The latent space provides a nearly perfect representation of the data, as the two components account for almost all of the variance in the selected variables. Farms plotted near each other exhibit similar profiles across these five variables.

The analysis of correlation ratios for categorical and continuous variables (Table 4) indicates that education and operation type are primary drivers for Component 1, suggesting that differences in farmer education and operation type shape this latent dimension. In addition, Farmers’ agency (Age and production experience) is an important driver for component 1. The farm size category is the most influential variable for Component 2 (i.e., farm size is a key determinant of this second latent dimension). Larger farms tend to score differently compared to smaller farms. The FAMD results produce a distinct two-dimensional map of farms when combined with the K = 4 cluster solution. The farms in this space exhibit common features (farmers’ age, experience, education, farm size, and operation type) (Figure 3). The identified latent structure helps to understand the factors that explain cluster differences and can inform the development of specific interventions and policy recommendations.

## 4. Discussion

The study revealed four unique clusters of producers whose make up varied according to their level of compliance and risk perceptions. It also showed that these differences in perception and practices influenced respondents’ attitudes towards endemic and foreign animal diseases. For instance, respondents’ implementation of enhanced biosecurity measures or improvement of their operations’ biosecurity were influenced by several normative factors such as different opinions of key stakeholders in the swine industry. The opinions of attending veterinarians and the swine producers’ association opinions were most influential. Previous studies have also reported that farmers regard veterinarians as their most trusted source of information on animal health and biosecurity measures [12,42,43]. Majority of respondents never felt any pressure from fellow producers to increase biosecurity measures in their operations. This finding does not align with experimental simulations (effects of social cues on biosecurity practices) reported by previous studies, where participants were more likely to comply with biosecurity when they saw others doing so, and showed an increased likelihood of noncompliance when they saw others violate biosecurity protocols. This further illustrates the complexity of biosecurity decision making, in situ as opposed to in silico where producers are faced with rapidly evolving scenarios and a myriad of competing interests [6,44].

Although some respondents believed that they implemented biosecurity measures on their farms, none of them practiced all the recommended steps of enhanced biosecurity according to the SPS plan. Biosecurity practices considered were the presence of a biosecurity manager, functional perimeter buffer area (PBA), vehicle cleaning /disinfection points external to the farm proper, an active and functional line of separation within the premises where internal biosecurity was strictly managed, and the monitoring and control of movements in and out of clean areas. Other practices included avoiding the sharing of equipment between farms, the ability to restrict access (lock) to farm buildings, and the presence of a backup plan in the event of the introduction of a FAD to the farm [15,20]. Our findings are in line with an extensive study conducted to evaluate biosecurity trends and practices in the US swine industry that showed a strong industry involvement in voluntary implementation of biosecurity measures but yet with several key gaps in compliance implementation of key biosecurity protocols, such as worker and visitor movement control and PPE use [6,37].

Both external biosecurity measures, actions taken to keep pathogens out, and internal biosecurity practices, measures taken to stop pathogens from spreading once the pathogens are in, are crucial in disease management, i.e., preventing introduction and spread to and from a given farm [45]. For certain diseases, such as PEDV, PRRS, influenza, which have multiple routes of transmission, including fomite/mechanical spread, the hygiene of vehicles is a crucial biosecurity component [5,20,46,47,48]. As such, the presence of disinfection/sanitization stations external to the farm and PBA minimizes the risk of introducing pathogens into the farm [45]. Moreover, internal practices to separate clean areas from dirty areas (LOS) and strict adherence to hygiene practices such as the use of protective clothing and showering in and out of these areas have been documented to considerably decrease the risk of disease spread and aid with biocontainment of pathogens [49,50].

While there are varied reasons why farms would choose to respond in a certain way to biosecurity threats, it is clear from this and previous studies [29] that certain trends exist, and specific factors may influence how biosecurity practices are implemented on US swine farms. According to previous studies, swine producers’ decisions to implement or not implement biosecurity measures are influenced by psychological (low risk perception and distrust of biosecurity recommendations), economic factors, and lack of clear risk communication (inconsistent messaging) [6]. First, an elaborate sensitization that enables producers to identify and implement the components of enhanced biosecurity measures would reduce the assumptions that producers are safe and implement enhanced biosecurity [20]. Although most of the respondents were confident about the health status of their herds, FADs may jolt producers into implementing more stringent biosecurity measures, as previous studies showed enhanced application of biosecurity measures following outbreaks of diseases [25,51].

However, without adequate understanding of, capacity to implement, and optimum compliance with the recommendations of the SPS, the US swine industry remains at risk if a new disease is introduced. Such an event would further strain biosecurity measures already challenged by endemic diseases. This ongoing pressure may partly explain why PRRS and PEDV are still prevalent. Thus, working with attending veterinarians would be ideal for optimal outreach, and engagement of these producers since their opinions were highly regarded, as seen in the present and previous studies [42,43]. Lastly, while the confidence of the respondents in their ability to address any health challenges in the production systems can be lauded, proper and regular biosecurity gap assessments would be beneficial to ensure that the true status is always known, and areas where more effort is needed to manage the risk are revealed [12,52]. Our study is, however, limited by the number of respondents and the proportional representation of the different production and operation types. Additionally, potential nonresponse bias and the underrepresentation of informal, small-scale, or backyard producers who may be less visible in formal databases or digital outreach could affect the generalizability of our findings. Future research should endeavor to collect more diverse data, including organic and backyard production systems, which may play a pivotal role in the case of disease outbreaks.

As previously mentioned, the cluster analysis produced four distinct profiles of swine producers who implemented biosecurity differently, showing diverse approaches to risk management in the swine industry. Previous studies by Laanen et al. [53] and Ribbens et al. [54] supports these findings as biosecurity adoption depends on several related factors such as farm characteristics and producer demographics, and risk perceptions. Among the clusters, Clusters A and B have a unique dichotomy among the younger, less experienced producers. Both groups had similar demographic characteristics, yet their risk perception profiles were different. Cluster A producers had high disease risk perception and maintained moderate biosecurity implementation across most measures, and even performed well in some practices, such as LOS and rendering. This indicates producers in these clusters may be selectively implementing biosecurity measures they perceive as practical or cost-effective. Farmers in this cluster were primarily involved in breeding operations, which were highly susceptible to disease transmission [20]. The gap between how producers perceive risks and how they implement risk management has been observed in livestock farms and could be attributed to factors such as financial constraints, technical limitations, or incorrect assessment of implementation benefit [43,55,56]. According to the transtheoretical model (TTM), farmers in this cluster seem to be in the preparation stage as they are actively practicing some biosecurity measures and are primed to implement more biosecurity measures [57].

On the other hand, Cluster B producers, who were also young and inexperienced, demonstrated variable biosecurity adoption patterns. This variability may be related to the resource constraints typical of smaller operations [29], leading to selective biosecurity implementation as in cluster A. Previous studies reported that smaller farms tend to choose specific biosecurity practices based on their assessment of cost-effectiveness instead of adopting complete protocols [20,58]. Thus, policy directions need to provide financial support or incentives such as low-interest rate loans to small-scale farmers as previously suggested [6,59]. Farmers in Cluster B are in the second stage of the contemplation stage, a second stage in the transtheoretical model [57]. They are aware and acknowledge the importance of implementing biosecurity in their farms, and they have also adopted a few measures, but they have yet to commit themselves to enhanced biosecurity measures [60].

Farms in Cluster C were characterized by moderate ages and production experience, mostly independent producers. This Cluster presented a concerning pattern with consistently low adoption across most biosecurity measures. This condition may reflect what psychologists refer to as “optimism bias,” where people become too optimistic and underestimate their risk [61], or it may also be due to normalization of higher mortality rates on their farms. A recent study also showed that swine producers who perceived a higher risk of FAD, such as African Swine Fever (ASF) were significantly more likely to invest in biosecurity measures compared to those with lower risk perception, which may also explain the mismatch between lower risk perception and higher mortality rate in farmers among farms in this cluster [59]. Similarly, in addition to resource limitations, skepticism about disease risk was identified as a major barrier to compliance with biosecurity practices [58,62]. Possibly resulting from their lack of trust in government agencies or limited confidence in the suggested strategy’s ability to prevent disease emergence and transmission. This disconnect suggests potential gaps in disease recognition, reporting accuracy (inadequate record-keeping systems), or management effectiveness that need further investigation to precisely identify the disconnect between perceived risk and the actual mortality rate. Farmers in this cluster belong to the precontemplation stage, the first stage according to the stages of change theory (transtheoretical model), as they are not yet considering adopting enhanced biosecurity measures, and they perceive that disease risk is low [57,60].

Cluster D comprised older, highly experienced farmers, along with more educated individuals with the highest level of compliance compared to the other clusters. The difference suggests systematic variation in biosecurity practices across clusters, potentially contributing to differences in mortality. Cluster D embodied the highest level of biosecurity implementation practices, maintaining below-average mortality rates despite managing complex, mixed-farm operations. Farms within this group may serve as role models for training and sharing best practices to improve biosecurity across the sector. Previous studies also reported that larger, more experienced operations were more likely to implement enhanced biosecurity protocols, potentially due to better access to resources [35]. Similarly, previous studies [33,62,63] showed that education and experience were important predictors of biosecurity compliance in poultry farms. Recently, Baye et al. [59] also reported that experience in the swine industry and knowledge of biosecurity has been identified as strong positive predictors of biosecurity implementation. The better performance of Cluster D farms could be attributed to the “learning by doing” concept in agricultural practices. Experienced producers have probably encountered disease outbreaks throughout their careers, which gives them practical knowledge of the economic consequences of inadequate biosecurity [42]. The combination of practical experience and formal education seems to result in better biosecurity practices. Producers in this cluster are in the Action/Maintenance stage, a fifth and final stage of change according to the transtheoretical model, as they have consistently shown the strongest uptake and highest adherence across all biosecurity practices assessed in this study [57,60]. Farmers in this cluster can be recognized for the best practices in biosecurity measures and used as peer mentors for other clusters in the lower stage of biosecurity adoption.

The FAMD results produced a distinct two-dimensional map of farms when combined with the K = 4 cluster solution, implying that the latent structure captures both an “educational/operational” dimension (Component 1) and a “scale” or “resource” dimension (Component 2). The nearly 100% variance explanation observed by the two-component solution shows that the selected variables effectively identify the main sources of variation among swine farms and help make targeted intervention strategies. The two distinct dimensions observed in Component 1 and Component 2 align with previous literature on agricultural economics and farm management that reports farmers’ education and operational specialization determine management practices and innovation uptake for sustainable agricultural practices [62]. The scale/resource dimension (Component 2) matches the established relationship between farm size and resource availability in agricultural production systems. Operations with larger sizes typically have better financial resources, access to technologies, and expertise. The dimensional separation indicates that biosecurity implementation depends on two distinct mechanisms: knowledge-based factors and resource-based constraints, factors that need to be taken into account when implementing biosecurity improvement projects.

## 5. Conclusions

The US swine industry has yet to achieve uniform compliance with the SPS biosecurity plan, as many producers believe their current disease prevention methods are adequate, even though their practices do not fully align with the recommended biosecurity measures. Cluster analysis and FAMD identified four producer clusters, which operate along two fundamental dimensions of variation that can help develop better biosecurity practices. The study shows that the adoption of biosecurity depends on producer characteristics and farm attributes within the swine industry. The identification of Cluster D as the “gold standard” group, characterized by experienced and educated producers with larger operations that have implemented enhanced biosecurity measures, confirms that human capital and resource availability are essential factors for successful disease prevention strategies. This study also identified two high-risk groups: Cluster A, which has an elevated risk perception but minimal biosecurity practices, and Cluster C, which has a low disease risk perception but a high mortality rate, indicating vulnerabilities that require immediate attention. The four clusters of farmers match the Transtheoretical Model (TTM) progression from precontemplation (Cluster C) for low adoption to action/maintenance (Cluster D), which indicates distinct levels of readiness for biosecurity practice adoption and maintenance. The stages of biosecurity adoption progress from awareness creation in precontemplation to practice reinforcement in action/maintenance through specific intervention strategies. Additionally, the FAMD results revealed factors that drive cluster differences and variations in the adoption of biosecurity practices—critical information needed for targeted intervention. The findings indicate that efforts to improve swine biosecurity should transition from uniform standardized interventions to differentiated, tailored methods by understanding producers’ heterogeneity. The identification of unique producer profiles provides a basis for applying precision agriculture principles to biosecurity management, with immediate practical applications; for example, cluster-specific profiles can serve as a guide for tailored intervention strategies. In the future, we suggest that longitudinal studies may help identify whether farms remain in their cluster or change as they gain experience, leading to the development of targeted support programs. This may include the addition of systems-based approaches such as Agent Based Modelling (ABM) to allow for dynamic simulations of farmers’ decision-making processes and their influence on disease transmission. Moreover, economic analyses linking cluster membership to profitability may strengthen the justification for investing in biosecurity. Factors affecting the adoption of biosecurity could be better understood by involving key information sources, such as veterinarians, who are most trusted by farmers. The findings of this study should be interpreted within the context of its limitations, such as the small sample size of respondents and the lack of adequate representation of production and operation types. Furthermore, we recommend validating the cluster structure with many farms across different regions of the US to enhance the generalizability of the patterns observed in this study. While we acknowledge the limited sample size and our limited ability to generalize these findings, the insights obtained from these producers offer valuable information about practices in a major swine production region of the U.S.

## Figures and Tables

**Table 1:** Standardized Median Scores for Numeric Variables Across Clusters. Each value represents the standardized median (z-score) for the listed variable within each cluster. Positive values indicate above-average levels (e.g., age, experience, risk perception), while negative values indicate below-average levels. For mortality, higher values represent greater reported mortality relative to the sample average.

Cluster	Farmers Age	Production Experience	Endemic AD Risk Rank	FAD Risk Rank	Mortality
A	−0.66	−0.84	1.48	2.33	0.04
B	−0.35	−0.84	−0.7	−0.81	−0.69
C	0.28	−0.49	−0.7	−0.13	0.95
D	0.67	1.22	0.03	−0.36	−0.51

**Table 2:** Distribution of Education Level Among the Clusters.

Cluster	College (%)	Grad/Professional (%)	High School (%)	Less than High School (%)
A	28.6	28.6	42.9	0
B	52.9	17.6	29.4	0
C	50	20	20	10
D	66.7	9.5	19	4.8

**Table 3:** Distribution of Production Type and Farm Size Across Clusters.

Cluster	Production Type (%)			Farm Size (%)			
	Contract Grower	Extensive/Outdoor	Independent Producer	Small	Medium	Large	Very Large
A	42.9	14.3	42.9	42.9	28.5	0	28.6
B	11.8	23.5	64.7	82.4	11.8	5.9	0
C	20	10	70	50	10	30	10
D	9.5	9.5	81	42.9	14.3	9.5	33.3

**Table 4:** Correlation Ratios (η^2^) for Categorical Variables in FAMD.

Variable	FAMD Component 1 (η^2^)	FAMD Component 2 (η^2^)
Education	0.59	0.37
Farm Size Category	0.19	0.74
Operation Type	0.59	0.62
Age	0.55	0.01
Production Experience	0.46	0.09

This table shows that education and swine operation type are the main factors influencing component one of the latent dimension. Equally high correlation ratios (η^2^ = 0.59). between the two variables in component one, may indicate farmers’ educational backgrounds and the type of swine operation they run may co-vary and define a major axis of variation in the dataset, possibly showing variation in management style, awareness, or adoption of biosecurity practices within or between different operation systems. The farm size is the key factor shaping component two of the dimensions; operation type also plays a significant role, as shown by a strong correlation ratio.

## Data Availability

The data presented in this study are available from the corresponding author upon reasonable request. Access is restricted due to confidentiality agreements and producer privacy concerns.

## Supplementary Materials

The following supporting information can be downloaded at: https://www.mdpi.com/article/10.3390/pathogens14111080/s1, Table S1: Survey item domains and response formats.
